# ß‐Hydroxy ß‐methyl butyrate, a muscle‐building supplement, restores neuronal plasticity in Alzheimer’s disease model

**DOI:** 10.1002/alz.087808

**Published:** 2025-01-09

**Authors:** Ramesh Kumar Paidi, Kalipada Pahan

**Affiliations:** ^1^ Rush University Medical Center, Chicago, IL USA; ^2^ Jesse Brown VA Medical Center, Chicago, IL USA

## Abstract

**Background:**

Alzheimer’s disease is the most dreaded multifactorial neurological illness for which there is currently no known treatment. Although the exact cause of AD is still unknown, several factors related to lifestyle, genetics, and environment are known to have a significant role in the disease’s development. Alzheimer’s disease is characterized by neuronal loss, neurofibrillary tangles, and senile plaques. Still, synapse loss is thought to be a major neuropathology associated with AD. β ‐hydroxy β ‐methyl butyrate (HMB) is a common muscle‐building supplement used by bodybuilders to enhance exercise‐induced increases in muscle development and strength. HMB is an extremely safe supplement that shows no negative effects even after prolonged use.

**Method:**

In primary mouse hippocampal neuronal cultures, we first validated the effect of HMB in terms of neuronal plasticity and neuronal markers. Additionally, we gave six‐month‐old XFAD animals oral HMB for 30 days, and we used the T‐Maze, Barnes Maze, Novel Object Recognition, and locomotion tests to evaluate the animals' cognitive skills. In vivo pathway inductions were analyzed using western blotting and immunohistochemical studies.

**Result:**

In the current study, HMB treatment prominently elevated neuronal plasticity markers like Glut N2A, Glu‐A1, Snap25, and PSD 95 in primary hippocampal cultures. HMB restores the cognitive malfunctions in 5xFAD AD mice; SNAP25 and PSD 95 expressions were increased following HMB administration. This was consistent with the phosphorylation of CREB and the increased calcium influx in the hippocampal region implicated in improving synaptic plasticity.

**Conclusion:**

Our results indicate that HMB increases the expression of synaptic proteins and controls CREB phosphorylation, which could regulate neural plasticity and lead to cognitive recovery in AD models. Given that HMB is a strong, safe supplement, more research on its potential benefits for treating AD seems needed.

